# Active or Healthy Ageing: “A Wonderful Journey between Body, Cognition and Emotion”

**DOI:** 10.3390/ejihpe12060039

**Published:** 2022-05-25

**Authors:** Gonçalo Dias

**Affiliations:** 1 Polytechnic Institute of Coimbra, Coimbra School of Education, Rua D. João III, Solum, 3030-329 Coimbra, Portugal; goncalodias@fcdef.uc.pt; 2 Research Unit for Sport and Physical Activity, University of Coimbra, 3004-531 Coimbra, Portugal; 3 Applied Research Unit (IIA), Polytechnic Institute of Coimbra, Robocorp, 3045-093 Coimbra, Portugal

This Special Issue, “Active or Healthy Ageing”, presupposes a dynamic balance between body, cognition, and emotion, and it promises to be an important resource for any professional working with the older adult population. In this sense, we begin our “*journey*” with “*The Relationship between Cognitive Status and Retained Activity Participation among Community-Dwelling Older Adults*” [1], by Fatemeh Adelirad, Maryam Moghaddam Salimi, Iman Dianat, Mohammad Asghari-Jafarabadi, Vijay Kumar Chattu and Hamid Allahverdipour. The study suggests that different types of activity may have various effects on balance and on some selective cognitive components in older people. On the other hand, Anna Koteneva, Tatiana Berezina and Stanislav Rybtsov, in the study entitled “*Religiosity, Spirituality and Biopsychological Age of Professionals in Russia*” [2], found that the religiosity of professionals increases with natural ageing and the deterioration of their physical condition, and this phenomenon does not depend on gender. Religiosity to a greater extent affects psychological age, the indicator of the psychobiological maturity of a professional, and, to a lesser extent, biological age. Most of the indicators of religiosity are inherent in a person who is more mature in psychobiological terms. Finally, the biological age of professionals increases due to asthenic experiences, while gaining faith in God, unusual religious experiences, and the existential meaning of life can reduce it. Moreover, “*Psychometric Properties of the Lasher and Faulkender Anxiety about Aging Scale (AAS) among Iranian Older Adults*” [3], by Amir Pakpour, Shamsedin Namjoo, Khadijeh Sabahiazar, Mohammad Asghari Jafarabadi, Vijay Kumar Chattu and Hamid Allahverdipour, indicated that the older adult population of society is exposed to multiple daily stressors, such as the loss of loved ones, dysfunctional mobility, financial dependence, and suffering from numerous chronic illnesses. The result of the CFA showed that this four-factor model had a good fit for the data. The findings were also confirmed by Rasch analysis. The authors conclude that Persian version of the AAS is valid and reliable for measuring ageing anxiety among older Iranian adults. Last, Mário Pereira, Rodrigo Mendes, Rui Mendes, Fernando Martins, Ricardo Gomes, José Gama, Gonçalo Dias and Maria António Castro, in the study entitled “*Benefits of Pilates in the Elderly Population: A Systematic Review and Meta-Analysis*”, concluded that the efficacy of Pilates has been shown in various areas of Healthy Ageing (HA) and has proven to be affordable and safe for the majority of people. Hence, future studies should focus on the analysis of the relationship between the cost and the benefit of a Pilates intervention in the elderly population to better understand how health costs can be minimized and to contribute to a multidisciplinary and generalized study of HA.

So, based on the studies mentioned in this *Editorial*, the number of people aged 80 years or more will triple and extend to 434 million by 2050. On the other hand, the number of people aged over 60 is increasing at a yearly rate of 3%. The prediction is that, in 2050, the elderly will represent 22% of the population! This demographic evolution has a strong impact and is an indicator of the social transformation of the 21st century. Thus, physical activity can play a crucial role in the protection against age-related morbidity and is crucial for older adult population. Accordingly, I hope that clinicians, therapists, exercise professionals, among others, that work with the elderly population may find a “viable strategy” towards healthy ageing, contributing to a healthier and more active ageing [4].

Given the above, in the future, I look forward to continuing this wonderful “*journey*”, together, with the important support of *Eur. J. Investig. Health Psychol. Educ*.
